# An German Short-Version of the “Sensory Perception Quotient” for Adults With Autism Spectrum Disorder

**DOI:** 10.3389/fpsyt.2022.781409

**Published:** 2022-06-14

**Authors:** Christoph Klein, Teresa Miczuga, Marie Sophie Kost, Hannah Röring, Tomasz A. Jarczok, Nico Bast, Ulf Thiemann, Christian Fleischhaker, Ludger Tebartz Van Elst, Andreas Riedel, Monica Biscaldi

**Affiliations:** ^1^Clinic for Psychiatry, Psychosomatics und Psychotherapy in Children Adolescents, Medical Faculty, University Hospital Freiburg, Freiburg, Germany; ^2^Clinic for Psychiatry, Psychosomatics und Psychotherapy in Children and Adolescents, Medical Faculty, University Hospital Cologne, Cologne, Germany; ^3^Second Psychiatry Department, National and Kapodistrian University of Athens, Medical School, University General Hospital “ATTIKON, ” Athens, Greece; ^4^Department of Child and Adolescent Psychiatry, Psychosomatics and Psychotherapy, Autism Research and Intervention Center of Excellence, University Hospital Frankfurt, Goethe-University, Frankfurt, Germany; ^5^Clinic for Psychiatry, Psychosomatics und Psychotherapy in Children and Adolescents, LVR Hospital, Bonn, Germany; ^6^Clinic for Psychiatry und Psychotherapy, Medical Faculty, University Hospital Freiburg, Freiburg, Germany; ^7^Clinic for Psychiatry, Medical Faculty, University of Luzern, Lucerne, Switzerland

**Keywords:** autism spectrum disorder, sensory features, Sensory Perception Quotient (SPQ), sex differences, adults, Autism Quotient, Empathy Quotient, IQ

## Abstract

Sensory features in autism spectrum disorder (ASD) have received increasing interest in clinical work and research during the recent years. With the Sensory Perception Quotient (SPQ), Tavasolli and colleagues have produced a self-rating scale for adults with ASD that measures sensory hyper-sensitivity in different sensory modalities, without also tapping cognitive or motivational aspects that precede or follow autistic sensory experiences. Here, we present the results of a translation of the SPQ to German and its short version as well as their validation in samples of autistic or neuro-typical participants. We, furthermore, present the psychometric properties and validities of Tavasolli's original SPQ-short version as well as an alternative short version based on different psychometric item-selection criteria. We can show here that our alternative SPQ-short version, overlapping with the original short-version in 61% of its items, exhibits superior reliabilities, reasonable concurrent validities with other related measures. It, furthermore, exhibits excellent differentiation between autistic and non-autistic samples, underscoring its utility as a screening instrument in research and a clinical instrument to supplement the ASD diagnostic process.

## Introduction

Qualitative impairments in social interaction and communication as well as restricted interests and repetitive behavior are the core symptoms of autism spectrum disorder (ASD) and have guided much of the ASD research so far. Since the publication of the Diagnostic and Statistical Manual—Version 5 (DSM-5), however, sensory features in persons with ASD have gained in research interest and are defined as “hyper- or hypo-reactivity to sensory stimulation as well as “unusual interest in sensory aspects of the environment” by the American Psychiatric Association (1). Recognition of sensory abnormalities in ASD has been incorporated already in influential theories about ASD, including the “weak central coherence” (2), “enhanced perceptual functioning” (3), the Intense World Theory (4), or Bayesian accounts of the disorder (5). It has been estimated that 30-100% of autistic individuals show sensory abnormalities. Given that sensory abnormalities have been considered as neural in origin, rather than being the consequence of dysfunctional sensory organs (6, 7), the question arises whether they are modality-specific or modality-unspecific. Weiland et al. (8), for instance, reported increased sensory sensitivity across various sensory modalities, except smell. Others, by contrast, have reported increased sensory sensitivity primarily in the auditory modality (9, 10). Sensory abnormalities, furthermore, may manifest in hypo-sensitivity in one sensory channel (e.g., pain or coldness) alongside hyper-sensitivity in other sensory modalities (e.g., aversions against certain noises or the surface structure of cloths) and may greatly differ between autistic individuals (11). A recent meta-analysis by Ben-Sasson et al. (10) has shown that hyper-sensitivity is a key feature of autistic sensory experience.

Sensory abnormalities may contribute to higher-order functions such as social cognition or may be even mistaken for them if, for instance, an autistic child does not respond when his/her name is called or reacts aversively to touch. They may also impact on non-social autistic features when, for example, repetitive behavior is a result of unusual seeking or avoidance of sensory stimulation. Repetitive behavior, distress as well as melt- or shutdown reactions in ASD can be associated with sensory hyper-reactivity. Understanding sensory abnormalities in autistic individuals may therefore deepen the understanding of the core symptoms of autism and its clinical heterogeneity (12).

Given that autism shows a male preponderance of diagnoses and autistic females can exhibit divergent core symptoms (13), another important distinction within the ASD population regarding sensory sensitivity is sex. Lai et al. (14) reported greater “unusual sensory responses”—a composite item created from three sensory items of the ADI-R—in female compared to male autistic individuals matched according to age and IQ. Likewise, Weiland et al. (8) found greater sensory sensitivity in female compared to male autistic individuals. More research into sex differences in sensory sensitivity is required to reach more definite conclusions.

Sensory features of autistic individuals are not always observable by others. Therefore, questionnaires have been designed to assess sensory features in adolescents or adults with autism, including the “Sensory Experience Questionnaire” [SEQ; (15)] or the “(Short) Sensory Profile” [(S)SP; (16)] for the evaluation of sensory abnormalities of children by their parents or the “Glasgow Sensory Questionnaire“ [GSQ; (17)], a self-rating scale of hypo- and hyper-sensitivity in all sensory domains. Also, the “gold standard” diagnostic instruments in childhood and adolescence, the Autism Diagnostic Schedule—Version 2 and the Autism Diagnostic Interview—Revision [ADOS-2 and ADI-R; (18, 19)] provide a few non-specific items regarding this symptom domain.

With the “Sensory Perception Quotient” (SPQ) by Tavassoli et al. (20, 21), a self-rating scale for sensory abnormalities in adults has been published. In contrast to other such self-rating scales, the SPQ items are meant to expel all cognitive-attentional or emotional-motivational contents preceding or following sensory experiences, thus focusing on sensory semantics specifically and providing purer construct validity. A short-version of the SPQ has been developed by Tavassoli et al. (20, 21) that consists primarily of items indicating hypersensitivity. Importantly, this short-version was created from the complete item pool by extracting the set of items constituting the first principal component of a factor analysis. While this procedure ascertains factorial validity of the short-version, it does not take into account group discriminability at the item level. In other words: this criterion of item selection, while being valid, does not take into account how the individual item(s) as well as their sum discriminate between groups. This is, however, important, if the correlation of questionnaire score and symptomatology is a prime validity criterion, like in the present case. Furthermore, a PCA with varimax rotation was employed to examine the factorial structure of the five sensory sub-scales (hearing, vision, touch, smell, taste). In addition, this PCA enabled comparisons of the relative sizes of the factor loadings of these sub-scales on factor 1 to identify a marker sub-scale of individual differences in sensory experiences (by virtue of its highest factor loading).

Based on a translation of the original SPQ (20, 21) to German, the present study pursued the following aims: Firstly, to determine the relative importance of hyper- and hypo-sensitivity for autistic sensory experiences. Secondly, to introduce an alternative short version of the SPQ that is maximizes statistical separation of autistic and neuro-typical groups. Thirdly, to determine psychometric consistency of items and sub-scales by use of Cronbach's alpha and factor analysis. Fourthly, to establish concurrent validity of the SPQ and its short version by correlations with important criteria like Autism Quotient (AQ), the Empathy Quotient (EQ), age and intelligence. The AQ is a 50-item self-rating scale for the assessment of autistic traits that comprises of the sub-scales social skills, attention switching, attention to detail, communication and imagination with good psychometric properties (22). The EQ is a 60-item self-rating scales that includes 40 items on empathy and, again, good psychometric properties (22). Fifthly, to investigate general and group-specific sex differences and precise them according to sensory modalities.

## Methods

Our study was approved by the ethics committee of the University of Freiburg (EK 85/16) and all study participants gave their informed written consent beforehand.

A total of *N* = 188 adult participants took part in the study, including N=85 autistic individuals with an autism spectrum diagnosis (age: 38.6 ± 13.3, 18-62 years; 65% male; IQ: 110 ± 16) and *N* = 103 healthy control participants (age*:* 36.5 ± 14.5, 19-76 years; 54% male; IQ: 112 ± 15). These groups did not differ significantly regarding sex [$\chi {{}^{\text{2}}}_{\left(1\right)}$ = 2.06, *p* = 0.151], age [*t*_(186)_ = −1.00, *p* = 0.318] and IQ [*t*_(150)_ = 0.71, *p* = 0.481]. The autistic participants were current or former patients treated in the Department of Psychiatry (*N* = 70) or the Department of Child and Adolescent Psychiatry (*N* = 17). Controls were recruited with flyers posted at public places within the city. General inclusion criteria were: age > 18.0 years, German as mother language, IQ > 70. For the autistic individuals, an autism spectrum diagnosis (F84.0, F84.1, or F84.5) was required; for controls, the absence of a psychiatric or neurological diagnosis. The diagnostic procedures included the ADOS-2 and, for the Department of Child and Adolescent Psychiatry, also the ADI-R (23) as well as the parents questionnaires FSK and SRS. In addition to the German version of the SPQ (see below), the autistic participants recruited through the Department of Psychiatry took a set of additional questionnaires, including the Australian Scale for Asperger's Syndrome (ASAS); the Autism-Spectrum Quotient [AQ; (24)], the Empathy Quotient [EQ; (25)], the Ritvo Autism Asperger Diagnostic Scale-Revised [RAADS-R; (26)], the Fragebogen zu sozialer Angst und sozialen Kompetenzdefiziten. [SASKO; (27)], the “Freiburg Questionnaire of linguistic pragmatics [FQLP, (28); the Bermond–Vorst Alexithymia Questionnaire, BVAQ-AB, (29)], and the Movie for the Assessment of Social Cognition test [MASC; (30)]. After fulfilment of the inclusion criteria was confirmed, participants were contacted via phone or letter and were subsequently informed about the study details. Testing duration was about 2 h including each 40 min for filling in the questionnaires, intelligence testing and administration of the Structured Clinical Interview for DSM-IV [Strukturiertes Klinisches Interview für DSM-IV; SKID-I; (31)]. Intelligence testing using the Culture Fair Test [CFT-20R; (32)] was not possible in all participants as some had, for instance, moved to another city. Questionnaires were filled in at home, the interview and the IQ test were administered in our department. The SKID-I revealed that about half of the autistic sample had a co-morbid affective disorders. These disorders are, however, not regularly associated with atypical sensory experiences (33).

As described in Tavassoli et al. (20, 21), the SPQ consists of 92 items covering 5 sensory modalities with 20 or 16 items each: Vision (33), Hearing (33), Touch (33), Taste (24), and Smell (24). Different kinds of perception are differentiated (such as pressure, pain, temperature and vibration of touch). All responses are given on a four-point Likert scale (0-3). For hypo-sensitivity and hyper-sensitivity, 43 or 49 items, respectively, are provided and items with negated hyper-sensitive semantics (negating wording, e.g., “I do *not* …”) are recoded. The SPQ total score varies between 0 and 276, with a low score indicating hyper-sensitivity. In addition to the total score, sum scores for specific sensory modalities can be computed.

The guidelines for trans-cultural research (34) guided the translation of the original SPQ from English to German, including the translation to German by a bilingual researcher, the back-translation to English by another bilingual researcher, as well as group discussions in case of discrepancies and consultations with experienced clinicians regarding the semantics of individual items.

In order to derive a short version of the German SPQ that separates our participants groups statistically at least as well as the full version, those 33 items were picked that differed between groups at least at the 5% level (*p* < 0.05) and indicated greater sensitivity (that is, lower scores) in the ASD compared to the TD group. About 50% of these items showed moderate (Cohen's d ≥ 0.5) or large (Cohen's d ≥ 0.8) effect sizes in separating the groups, *the other ones small effect sizes (Cohen's d* ≥ *0.2)*. To determine (concurrent) validity of the SPQ, its sum score was correlated with those of the AQ and EQ. Any outcome of such correlations will help understand how the SPQ sum scores may fit into a nomological network of inter-dependencies of constructs central to ASD.

The statistical analyses included univariate analyses of variance (ANOVA) with GROUP (autistic, controls) and SEX (male, female) as between-subject factors, *t*-Tests, correlation analyses using Pearson's r, and the estimation of Cronbach's alpha for reliability analyses.

In contrast to the procedures applied by Tavassoli et al. (20, 21), selection of items for a short-version of the SPQ was not based on factor analysis but on the power of the individual items to discriminate the autistic from the control group. This also included the analysis of item difficulties, item scatter and the consideration of minima and maxima in both groups separately (not reported here). See footer of Table 1 for the included items.

**Table 1:** SPQ group and sex differences.

	**Descriptive statistics**						**Group**			**Sex**		
	**ASD_**tot**_**	**TD_**tot**_**	**ASD_**f**_**	**ASD_**m**_**	**TD_**f**_**	**TD_**m**_**	**F_**1, 184**_**	***P***	**eta^**2**^**	**F_**1, 184**_**	***P***	**eta^**2**^**
SPQ_total_	103 ± 30^a^	119 ± 20	95 ± 32	107 ± 29	121 ± 18	118 ± 21	24.57	0.000	0.118	1.72	0.19	0.009
												
SPQ_Hearing_	26 ± 7	30 ± 5	25 ± 7	26 ± 7	30 ± 4	30 ± 5	24.49	0.000	0.117	0.20	0.66	0.001
SPQ_Touch_	17 ± 7	22 ± 7	15 ± 8	18 ± 7	23 ± 7	22 ± 7	25.56	0.000	0.122	1.19	0.28	0.006
SPQ_Vision_	26 ± 7	30 ± 5	25 ± 7	27 ± 2	31 ± 5	30 ± 5	24.02	0.000	0.115	0.96	0.33	0.005
SPQ_Taste_	16 ± 7	19 ± 5	14 ± 7	18 ± 6	19 ± 5	19 ± 6	8.40	0.004	0.044	3.89	0.05	0.021
SPQ_Smell_	17 ± 9	19 ± 6	16 ± 9	18 ± 9	19 ± 6	18 ± 6	3.42	0.07	0.018	0.47	0.50	0.003
												
${\text{SPQ-S}}_{\text{Tav}}^{\text{b},\text{d}}$	44 ± 17	56 ± 13	39 ± 17	47 ± 17	57 ± 12	56 ± 14	36.00	0.000	0.164	2.59	0.11	0.014
${\text{SPQ-S}}_{\text{DE}}^{\text{c},\text{d}}$	44 ± 13	62 ± 10	38 ± 12	46 ± 13	63 ± 9	61 ± 12	127.44	0.000	0.409	3.34	0.07	0.018

*ASD, Autism Spectrum Disorder; TD, Typically Developed (Controls); tot, total sample; f, female; m, male*;

a *:means ± standard deviation are displayed in columns*;

*^b^: Items: 2, 7, 12, 14, 19, 21, 31, 32, 33, 35, 36, 38, 42, 43, 45, 55, 58, 59, 60, 61, 62, 63, 68, 71, 73, 74, 75, 81, 84, 85, 87, 88, 89, 90, 91*;

*^c^: Items: 9, 11, 12, 17, 19, 23, 24, 26, 28, 31, 32, 33, 35, 37, 45, 55, 57, 59, 60, 61, 62, 64, 68, 73, 74, 75, 80, 82, 83, 84, 85, 88, 90*;

*^d^: Overlapping items of the two short-versions are underlined*.

## Results

### Group Differences, Sex Differences, and Sensory Modalities

As can be seen in Table 1, the group of autistic individuals showed a significantly lower SPQ total score when compared to controls, pointing to *increased sensory sensitivity*. This difference corresponds to a medium effect size of eta^2^ = 0.118 (corresponding to d = 0.73^1^ Looking at the specific sensory modalities, it becomes evident that this overall significance is mainly due to the modalities hearing, touch and vision (eta^2^ ≥ 0.115), less to taste, and not to smell (0.018 ≤ eta^2^ ≤ .044). Among the 20 items of each of the modalities hearing, touch and vision, 11-12 items show significant group differences; this holds only for 3 or 1 item(s) of the modalities taste and smell, respectively (not further detailed here). Overall, the inclusion of items from the modalities taste and smell *reduce* the diagnostic sensitivity of the overall scale.

While the overall sex differences are largely non-significant (see Table 1), significant GROUP x SEX interactions for the SPQ-total score (F_1,184_ = 4.472, p = 0.036, eta^2^ = 0.024) as well as the subscale scores for touch (F_1,184_ = 4.11, *p* = 0.044, eta^2^ = 0.022), taste (F_1,184_ = 4.39, *p* = 0.037, eta^2^ = 0.023) and, as a trend, vision (F_1,184_ = 2.89, p = 0.091, eta^2^ = 0.015) alongside the descriptive statistics shown in Table 1, point to greater overall and modality-specific sensory sensitivity in female as compared to male autistic individuals. No such sex differences are found for neuro-typical controls. The significant GROUP x SEX interaction thus results from overall and modality-specific sensory sensitivity in autistic, but not neuro-typical, females compared to males. Only with regard to hearing (F_1,184_ = 1.37, *p* = 0.24, eta^2^ = 0.007) and smell (F_1,184_ = 1.65, *p* = 0.20, eta^2^ = 0.009), sex differences do not differ between the autistic and non-autistic groups.

The original and present short-versions of the SPQ all exhibit better group effect sizes than the total score (see Table 1). This holds for the 35-item short-version produced by Tavassoli et al. (20, 21) (eta^2^ = 0.164) and even more so for the 33-item short-version derived in the present study (eta^2^ = 0.409). Both short-versions also confirm greater sensory sensitivity in female as compared to male individuals with autism, with the original short-version showing a slightly smaller (F_1,184_ = 2.995, *p* = 0.085, eta^2^ = 0.016) and the present short-version a clearly larger (F_1,184_ = 7.22, *p* = 0.008, eta^2^ = 0.038) GROUP x SEX interaction than the SPQ total score (see Table 1).

For the interpretation of the psychometric differences between Tavassoli's original short-version and the present one regarding overall group differences as well as group-specific sex differences, it is important to take a look at the composition of the short-versions regarding item contributions from the different sensory modalities. The original short-version includes 5, 6, 10, 10, 4 items from the modalities hearing, vision, touch, smell and taste, respectively, the present short-version includes 11, 11, 9, 3, and 1 items from these modalities. There is thus a preponderance of three of the four modalities that separate groups (hearing, vision, touch and smell) and three modalities that reveal group-specific sex differences at the statistical trend level or better (touch, taste, vision). Despite these differences in item composition between the original and the present short-version of the SPQ it is noteworthy that 61% of the 33 items of the present version are also included in Tavassoli's short-version (see footers a-c of Table 1).

### Reliability and Dimensionality

The reliabilities as expressed through Cronbach's alpha are for all three versions good to excellent for the ASD group and acceptable to good for the neuro-typical group (see Table 2). All three versions underline the psychometric homogeneity of the different SPQ item pools. Regarding sub-scales, a Principal Component Analysis (PCA) with varimax rotation and Scree Test for the pooled or separated groups led to the extraction of a single factor, hearing, that accounted for 63.4, 53.2, and 67.3% of the inter-individual variance in both groups pooled, or neuro-typical and autistic samples separately, respectively. Factor loadings of the sensory sub-scales are presented in Table 2.

**Table 2:** Factor loadings of sensory sub-scales on PCA factor 1.

	**Hearing**	**Vision**	**Touch**	**Smell**	**Taste**
Both groups	0.73	0.78	0.81	0.84	0.81
ASD only	0.75	0.82	0.83	0.85	0.84
TD only	0.59	0.62	0.77	0.86	0.78

### Convergent Validities

In controls, individual differences in the sensory scales are almost uncorrelated with IQ (Table 3), with correlations ranging between |0.00| and |0.08| and thus corresponding to very small effect sizes (Cohen's d ≤ 0.17). The small-to-medium negative correlation with age, however, suggests that with increasing age, sensory sensitivity increases too. Furthermore, in controls there are only negligible correlations between SPQ scores and AQ or EQ scores. The only exception here is the correlation of −0.21 or 0.21 between our short-version and the AQ and the EQ, respectively. These small-to-medium correlations indicate that increased sensory sensitivity is associated with increased autistic features and decreased empathy in control persons.

**Table 3:** Convergent validities and reliabilities of the different SPQ versions.

	**ASD**						**TD**				
	**AQ**	**EQ**	**Age**	**IQ**	**Cron-α**		**AQ**	**EQ**	**Age**	**IQ**	**Cron-α**
SPQ_total_	−0.39^***^	0.05	−0.20	0.02	0.92		0.05	−0.08	−0.18	0.08	0.82
SPQ-S_Tav_	−0.42^***^	0.07	−0.22^*^	−0.00	0.91		0.06	0.00	−0.20	0.01	0.82
SPQ-S_DE_	−0.43^***^	0.07	−0.23^*^	0.04	0.85		−0.21^*^	0.21^*^	−0.17	0.02	0.77

* *p < 0.05*;

*** *p < 0.001*.

For the autistic group, the correlations with IQ and age are similar to those of controls: Sensory abnormalities are not associated with intelligence and tend to increase with increasing age (Table 3). In the autistic group, the correlations with AQ and EQ reveal Increased sensory sensitivity is associated with greater autistic traits and somewhat (almost negligible) with greater empathy. In fact, all sensory modalities except hearing (r = −0.10) showed significant and substantial correlations (0.35 ≤ r ≤ 0.38) with the AQ score in the autistic group.

## Discussion

The present study translated the English “Sensory Perception Quotient” [SPQ; (20, 21)] to German and derived an optimized short-version of the scale. The main findings of this study are: (1) Hyper-sensitivity: Autistic adults show increased sensory sensitivity; (2) *New short version: A* 33-item short-version of the SPQ could be extracted that separates autistic from non-autistic participants with very large effect sizes and high internal consistency, being composed mainly of items from the sensory modalities hearing, touch and vision, measuring mostly hypersensitivity; (3) Consistency*: The* auditory modality is suggested as a marker scale of sensory hypersensitivity for both groups; (4) *Correlates: The* sensory experiences tapped by the SPQ are unrelated to intelligence but increase somewhat with age and show group specific correlations with autistic traits (AQ) and, less so, with empathy (EQ); (5) *Sex differences: Sex* differences were found exclusively for the autistic group, suggesting that female autistic individuals are more sensitive than male in the sensory modalities touch, vision and taste, but not hearing and smell.

Ad (1)—*Hypersensitivity:* With our German translation and adaptation of the SPQ it was possible to find sensory hypersensitivity in adult autistic individuals. This finding replicates previous reports on sensory abnormalities in autism using other questionnaires, including the Sensory Overresponsivity Scale [SensOR Scale; (20, 21)] as well as the Adult/Adolescent Sensory Profils [AASP; (35)]. Sensory hypersensitivity, known by clinicians to be characteristic (but not specific) of patients with autism, can therefore be considered as subjectively accessible by appropriate rating scales. This should hold, in principle, also for sensory hyposensitivity, which is, however, clinically less salient and not covered by this questionnaire. Our study has shown that using the item set provided by the SPQ and its German translation, autistic sensory experiences differ from non-autistic sensory experiences mainly in hypersensitivity in the domains audition, vision and touch. In this way, our findings basically agree with results of the recent meta-analysis by Ben-Sasson et al. (10) who have underscored that hyper-sensitivity is the most prominent dimension of autistic sensory experience.

*Ad (2)—New short version:* Regarding the differentiation of autistic and non-autistic individuals, all short-versions performed better than the long version of the scale. In the present study, this holds both for Tavassoli et al. (20, 21) factor-analytically extracted item subset but even more so for our item selection that was based on the individual items' group differentiation. Here, we found that 54 of the 92 SPQ items did not differentiate our autistic vs. non-autistic groups significantly. Overall, for the German version of the SPQ we found clearly better group separation (Cohen's d) than for the original English version (SPQ total: |0.66| vs. |0.28|; short-version by Tavassoli: |0.82| vs. |0.30|). This is presumably best explained *via* sample differences, be they language-, sampling- or in a broader sense culture-related. The item sub-set selected in the current study consists mainly of items from the modalities hearing, touch and vision, whereas taste and smell are represented by 3 or 1 item(s) only, respectively. This clear under-representation of smell in particular also replicates previous findings, as items from this modality did not differentiate groups in the studies of Tavassoli et al. (20, 21), Taylor et al. (36) and Weiland et al. (8).

Ad (3)—*Consistency:* Our reliability analyses focusing on Cronbach's alpha suggest good to excellent consistencies in the autistic group and acceptable to good consistencies in the control group, alongside slightly lower coefficients in our short-version. This pattern indicates that the different SPQ versions assemble item sets that map individual differences in sensory experiences in highly consistent manners, both in autistic and non-autistic groups. These different versions include the full (German) version of the SPQ (with subsets of items that do or do not differentiate groups), Tavassoli and colleagues short-version (based on item selection by factor analysis), and our short-version (based on item selection by group differentiation). Overall, this finding is complemented by factor analytical dimensional reduction, extracting consistently the auditory modality both within and across the diagnostic groups. More specifically: The results of the PCAs for both groups showed that inter-individual differences in the auditory domain explain more than 70% of the entire inter-individual variance in sensory experiences across all five modalities in the present samples. It has been suggested that autistic individuals are particularly sensitive in the auditory modality (9, 10). Finding here, however, that the auditory sub-scale is a marker of sensory sensitivity also in controls, suggests that the auditory modality might be amenable to individual differences in sensory sensitivity for reasons that are unrelated to the autistic condition. Humans orient in space and time primarily using the visual and auditory senses, but can select information at the sensory organ level only through the eyes. It is therefore tempting to speculate that the relative “passivity” of information processing through the ears renders this sensory channel relatively “susceptible” to individual differences in sensory sensitivity. It could be argued, furthermore, that the SPQ items for the visual modality tap more “sensory interests” and “hyper-acuity” whereas those for the auditory modality contain “purer” sensory content.

Ad (4)—*Correlates:* Our short-version and the one developed by Tavassoli et al. (20, 21) performed similarly in the concurrent validation with the AQ. Although the correlation of the present short-version with the AQ of r = 0.47 is clearly above the coefficients of r ≈ 0.14 (20, 21), r = 0.27 (36) or τ = −0.13 (8), the German version of Tavassoli's short-form and our short-form showed almost identic correlations with the AQ. This pattern of results suggests a greater influence of the participant sample rather than the item sample on the validity coefficients. This seems to hold, however, only for our autistic sample, as the control sample of our study showed similar correlations like the control and the autistic groups in Tavassoli et al. (20, 21). Still a different pattern was reported by Weiland et al. (8) in Dutch samples, in which the control group (τ = −0.23) showed a slightly higher correlation between SPQ and AQ than the autistic group (τ = −0.13). Altogether and except for the autistic group of the current study, the SPQ-AQ correlation appears to some sample-related volatility on an overall low level. Associations between sensory abnormalities and autistic traits have been reported in further studies, including studies with autistic children (37, 38), autistic adults (20, 21, 24) and community samples (17). No such correlations, however, were reported by Crane et al. (9). Regarding the EQ, correlations with the SPQ versions were very low and not exceeding |0.07| in our autistic group. Altogether, the AQ and EQ validity coefficients suggest that sensory abnormalities and core autistic traits as assessed with the AQ are indeed associated, but empathy is not.

Near-zero correlations of the SPQ were also found for IQ, confirming results reported by Tavassoli et al. [(20, 21); *p* = 0.95] and indicating that at least in the upper IQ range (present study: controls > 73, autists: > 83), sensory experience and cognitive abilities are unrelated features. This result is complemented by studies showing sensory abnormalities in low-functioning autistic individuals (39, 40) as well as studies with infants (41) and children (42, 43) that revealed no associations between sensory abnormalities and general or language- and cognition-related developmental levels. The three last-mentioned studies, however, applied the Mullen Scales of Early Learning (44) which tests in addition to receptive and expressive language also visual reception as well as fine and gross motor control and is therefore not a proper IQ test. By contrast, Crane et al. (9) reported large negative correlations of r = −0.66 and r = −0.44 for their above-average intelligent autistic and control individuals, respectively, between sensory sensitivity and just the Wechsler performance IQ, with correlations below 0.1 for the verbal IQ. Overall, and with the exclusion of the study by Crane et al. (9), results suggest that sensory abnormalities and intellectual capabilities are unrelated.

We found a moderate negative correlation between SPQ scores and age, suggesting a tendency for increasing sensory sensitivity with increasing age in both autistic and non-autistic individuals. Such findings, however, were not reported by Crane et al. [(9); r ≤ −0.17], Tavassoli et al. [(20, 21); *p* = 0.58], Taylor et al. [(36); r = 0.06] and Weiland et al. [(8); r = 0.06] for adults, and by Baranek et al. (45) and Kern et al. (46) for children. Given the rather moderate level of correlations for the different SPQ versions (r ≤ |0.22|) the discrepancy of our results should not be overemphasized.

Ad (5)—*Sex differences:* Sex differences in sensory sensitivity had already been reported by Weiland et al. (8) for their autistic sample only, using the Dutch short-version of the SPQ. In the present study that is based on a considerably smaller sample than the Dutch one, we could replicate such autism-specific sex differences for the entire scale and, in addition, break it down according to sensory modalities, showing increased sensitivity in females primarily in touch, vision and smell. These modality-specific sex differences together with the modality-specific group differences (autism vs. controls) would suggest that sensory hyper-sensitivity, while being considered a central nervous rather than a peripheral sense-organ phenomenon (7), may nevertheless not reflect a global CNS property but maybe confined to particular sensory functional systems of the brain.

Overall, here we have presented an alternative short-version of Tavassoli and colleagues' SPQ that performs better than Tavassoli's short-version regarding the differentiation of autistic and non-autistic groups and in a modality-specific manner the sexes within the autistic sample, performs similarly well regarding different validity criteria, and performs slight worse in terms of internal consistency.

## Limitations and Conclusions of the Present Study

The sample size of this study is only modest, which impacts the generalisability of our findings. Nevertheless, most effects or associations reported here were moderate to large in size, underlining the general robustness of our findings. Related to the first limitation, the set of additional measures used for concurrent validity analyses is rather limited and includes IQ, age, AQ and EQ. Positioning sensory features in a broader net(work) of inter-dependencies would be desirable but would require large sample sizes for appropriate modelling (e.g., through Structural Equation Modelling). The final short version presented here consists exclusively of items measuring perceptual hyper-sensitivity and this confined to the modalities touch, hearing and seeing. Future studies on this topic should therefore invest in building an item pool for the modalities smell and taste as well. In order to assess the utility of the scale for differential diagnostic purposes, it would be important to administer it to clinical populations other than the autistic that may also show sensory features, such as schizophrenia or ADHD.

Generalisability of findings, however, is not only a matter of sample size but also of sample composition. The results of the present study yielded in part clearly larger sizes of effects or associations compared to other studies using a short version of the SPQ which, as such, cannot be explained by sample sizes (i.e., degrees of freedom) *per se*. Some of these differences have been interpreted in the sense of sample and / or cultural differences and lead to the conclusion that more diverse samples should be recruited and administered the SPQ or already collected data sets should be concatenated to analyse sample and / or cultural differences meta-analytically. Other differences can be explained by differences in the methods of item selection for constructing a short version. This holds in particular for the item selection by statistical group separation (here) as opposed to factor analysis (Tavassoli and others). While the original short version of the SPQ (20, 21) separated our groups better than the original long version (see Table 1), the present short version outperformed even the original short version in distinguishing between autistic and non-autistic samples. We may therefore conclude that the present short version of the SPQ is excellently suited for screening purposes in studies on autism but also as an auxiliary diagnostic instrument for autistic patients as long as hyper-sensitivities in touch, hearing or vision are concerned.

Overall, our study has added to the field of autism research another translation of the SPQ by Tavassoli et al. (20, 21), a new short version of that scale that optimizes the separation of autistic and neuro-typical samples. Furthermore, our study underlines the importance of hyper- (rather than hypo-) sensitivity in autistic hearing, vision and touch and adds to the growing evidence of sex differences herein. While atypical sensory experience has become an established feature of the autistic symptomatology, it is probably by no means specific to this disorder.

## Data Availability Statement

The raw data supporting the conclusions of this article will be made available by the authors, without undue reservation.

## Ethics Statement

The studies involving human participants were reviewed and approved by the Ethics Committee of the University of Freiburg (EK 85/16). Written informed consent to participate in this study was provided by the participants' legal guardian/next of kin.

## Author Contributions

AR, MB, and CK planned the study. TM, MK, and HR collected and analyzed data. CK analyzed data and wrote manuscript. AR, MB, LT, CF, TJ, UT, and NB contributed to writing the paper. All authors contributed to the article and approved the submitted version.

## Conflict of Interest

The authors declare that the research was conducted in the absence of any commercial or financial relationships that could be construed as a potential conflict of interest.

## Publisher's Note

All claims expressed in this article are solely those of the authors and do not necessarily represent those of their affiliated organizations, or those of the publisher, the editors and the reviewers. Any product that may be evaluated in this article, or claim that may be made by its manufacturer, is not guaranteed or endorsed by the publisher.

## Footnotes

^1^ https://www.psychometrica.de/normwertrechner.html
